# Exploring evidence-policy linkages in health research plans: A case study from six countries

**DOI:** 10.1186/1478-4505-6-4

**Published:** 2008-03-11

**Authors:** Shamsuzzoha B Syed, Adnan A Hyder, Gerald Bloom, Sandhya Sundaram, Abbas Bhuiya, Zhang Zhenzhong, Barun Kanjilal, Oladimeji Oladepo, George Pariyo, David H Peters, Future Health Systems: Innovation for Equity

**Affiliations:** 1 Department of International Health, Johns Hopkins University Bloomberg School of Public, Baltimore, USA; 2 Institute of Development Studies, University of Sussex, Brighton, UK; 3 ICDDRB: Centre for Health and Population Research, Dhaka, Bangladesh; 4 Chinese Health Economics Institute, Beijing, China; 5 Indian Institute of Health Management Research, Jaipur, India; 6 University of Ibadan, College of Medicine, Faculty of Public Health, Ibadan, Nigeria; 7 The Institute of Public Health, Makerere University, Kampala, Uganda

## Abstract

The complex evidence-policy interface in low and middle income country settings is receiving increasing attention. Future Health Systems (FHS): Innovations for Equity, is a research consortium conducting health systems explorations in six Asian and African countries: Bangladesh, India, China, Afghanistan, Uganda, and Nigeria. The cross-country research consortium provides a unique opportunity to explore the research-policy interface. Three key activities were undertaken during the initial phase of this five-year project. First, key considerations in strengthening evidence-policy linkages in health system research were developed by FHS researchers through workshops and electronic communications. Four key considerations in strengthening evidence-policy linkages are postulated: development context; research characteristics; decision-making processes; and stakeholder engagement. Second, these four considerations were applied to research proposals in each of the six countries to highlight features in the research plans that potentially strengthen the research-policy interface and opportunities for improvement. Finally, the utility of the approach for setting research priorities in health policy and systems research was reflected upon. These three activities yielded interesting findings. First, developmental consideration with four dimensions – poverty, vulnerabilities, capabilities, and health shocks – provides an entry point in examining research-policy interfaces in the six settings. Second, research plans focused upon on the ground realities in specific countries strengthens the interface. Third, focusing on research prioritized by decision-makers, within a politicized health arena, enhances chances of research influencing action. Lastly, early and continued engagement of multiple stakeholders, from local to national levels, is conducive to enhanced communication at the interface. The approach described has four main utilities: first, systematic analyses of research proposals using key considerations ensure such issues are incorporated into research proposals; second, the exact meaning, significance, and inter-relatedness of these considerations can be explored within the research itself; third, cross-country learning can be enhanced; and finally, translation of evidence into action may be facilitated. Health systems research proposals in low and middle income countries should include reflection on transferring research findings into policy. Such deliberations may be informed by employing the four key considerations suggested in this paper in analyzing research proposals.

## Introduction

The interface between evidence and policymaking is complex, particularly in low and middle income countries and has received increasing attention in the literature [1]. Recent calls have been made for continued researcher engagement in exploring the interface [2-4]. The non-linear nature of translation of evidence into policy has been acknowledged and the multiple inputs into policy making processes in these settings have been subject to increasing review [5]. Decision makers and researchers often come from different cultures and have disparate motivations; thus priorities emanating from these two groups are often distinct [6,7]. Increasing the global knowledge base on the operation of the interface, especially in low and middle income countries, is required in order to facilitate evidence based health systems development.

Future Health Systems (FHS): Innovations for Equity is a research consortium that hopes to enhance understanding of the evidence-policy interface in the development of future health systems, with a particular focus on the poor [8]. The overall goal of the 'research to policy' thematic activities in the consortium is to understand the relationship between evidence and development of policies, especially their impact on the poor [9]. More specifically the consortium seeks to: document previous *experiences *of decision makers with health research; understand overall *values *placed on health research and evidence by decision makers; define the context and conditions under which decision makers will *demand *health research; identify *characteristics *of health research that make it attractive to decision makers; and explore the existence and performance of *institutional mechanisms *that allow interaction between research evidence and policy development and implementation at national and sub-national level [8].

The aim of this paper is to report on initial consortium efforts in strengthening the interface between planned health systems research and policy-making. The paper firstly describes essential context from the FHS country team research plans. Second, a description of the process utilized by the consortium to develop an approach to strengthening research to policy is provided. Third, four key considerations for evidence-policy strengthening are discussed. Fourth, findings from the six-country research agenda analyses based on these considerations are presented both at country level and across countries. Lastly the paper reflects on the utility of such an approach for health systems and policy research.

## FHS country team research projects – essential context

FHS is working in six low-to-middle-income countries, namely Bangladesh, India, China, Afghanistan, Uganda, and Nigeria. Consortium activities are focused on a "research partnership model" similar to that suggested by Costello and Zumla [10]. Each country team developed and completed a concept paper that will guide activities for the remaining duration of a five-year project. These concept papers propose empirical work to be conducted in the countries inclusive of issues relevant to the research-policy interface. A description of the work being conducted in each of the six countries, which is essential for understanding the concepts proposed in the paper, is presented below; further details are available from the FHS website [8].

The health system in Bangladesh has experienced significant reforms in the last decade with mixed public reactions [11]. A significant proportion of the poor in Bangladesh use informal health care providers as their first line of care, and this has increased since the reforms [11,12]. The general objective of FHS work in Bangladesh is to understand this informal care system and its interaction with the formal health system and local governance in Chakaria, a rural area of Bangladesh. The project aims to answer research questions focused on the: role of the informal health care system in influencing the health status of the poor in rural Bangladesh; defining the relationship between informal and formal health sectors; documenting health care utilization patterns, associated costs and their determinants; assessing service quality provided by informal health care providers; and exploring the role of elected local government representatives in health issues, particularly in relation to the poor. Study findings will then be used to develop, implement, and evaluate appropriate interventions to improve the health of the poor.

Two fundamental problems of the Indian health care system are that resources flowing through public administrative channels do not necessarily benefit the poor; and the population (the poor in particular) remains significantly unprotected against the burden of treating unanticipated major ailments [13]. FHS work in India will be conducted at the district level in West Bengal, in two phases. Phase I studies in the first year will prepare a knowledge base for development of equitable health care systems. The three principle research questions addressed in this phase are: 1) how links between poverty and health are manifest in the Indian health care market; 2) how oriented is the supply side environment towards equitable distribution of resources; and 3) does decentralization of institutions make systems work for the poor? Phase I will constitute: household surveys in three representative districts of West Bengal; an assessment of private and public providers through survey of selected facilities in these districts; and an assessment of selected decentralized institutions to identify strengths and weaknesses in implementing and overseeing pro-poor strategies. Phase I results will be used to develop a master proposal for a detailed pilot intervention plan in one district in phase II.

The transition from a planned to market economy and urban-rural disparities provide a unique context for studying health system development in China. The Chinese population of 1.3 billion people consists of 800 million rural dwellers. 87% of this rural population is not covered by any form of health insurance and nearly 100 million live below the poverty line [14]. Since 2003, China has established the New Cooperative Medical Scheme (NCMS) in rural areas with the county as the basic unit. Financing is from individuals, local government and central government pools; participation is voluntary. The NCMS has already covered over 20% of all counties in China, with over 70% individuals covered in these areas, and the Ministry of Health plans to expand coverage rapidly in the next few years. Two key challenges facing NCMS (re-design of financing and reimbursement mechanisms; regulation of quality and efficiency of healthcare providers) will be the focus of country work that combines literature review, policy research and field study. Three provinces from eastern, central and western China respectively will be chosen and investigation will be conducted in two or three counties in each province.

Maternal health is among the worst in the world in Afghanistan [15]. FHS work aims to analyze individual, household, and community vulnerabilities which influence maternal health service utilization. Key questions to be addressed are: what individual, household, and community factors influence *capacity *to utilize maternal health services; how do these factors interact to inhibit (or enable) maternal health service utilization; what are the effects on maternal health service use of rapidly changing institutional actors and processes in this context; what are the features of interventions which can influence these factors and increase appropriate maternal health care-seeking; and how can interventions take advantage of existing institutional arrangements or engender different institutional arrangements to enable maternal health care seeking? Four provinces will be selected based on the presence of at least one referral-level facility capable of delivering comprehensive essential obstetric care. Districts in these provinces that have public health facilities with capacity to deliver basic essential obstetric care will be identified. The work will include research methods such as key informant interviews and focus group discussions with a wide array of respondents. Findings will be used to develop a major intervention project to encourage appropriate maternal health service utilization in the country.

FHS will work at the national level and in two regions of Uganda. The two key research questions are: 1) What mechanisms can help policy makers get better value in terms of access, cost, volume and quality of services for the poor from both public and private not for profit providers?; and 2) How can users be empowered to demand quality services? The first year of work will consist of formative research to lay foundations for later years including: an extensive literature review covering the socioeconomic profile of health service users, health service quality, and community empowerment mechanisms to demand better services; secondary data analysis of the Uganda National Household Surveys, providing understanding of the available access to services for various population groups with emphasis on the poor; and qualitative approaches to understanding community perceptions on current service quality and mechanisms for improvement (especially community empowerment mechanisms). The second year will comprise a cross sectional study aimed at assessing the cost, volume and quality of services provided by public and private facilities. The third year will focus on instrument development to assess service quality in public and private health facilities. Thereafter innovative approaches to improve service quality in these facilities and mechanisms to empower the community to demand better services will be piloted for two additional years.

Malaria is a major cause of mortality and morbidity in Nigeria and is intricately linked with poverty; effective malaria treatment is essential to reducing this disease burden. Currently, sub-standard anti-malarial drugs thrive in unregulated drug markets and private sector services, and are used particularly by the poor. The goal of FHS research is the generation of knowledge necessary to design innovative interventions for effective malaria treatment, focused on the poor. The specific objectives are to: define patterns of poverty and vulnerability to malaria; document concerns of the poor regarding malaria treatment and access to information; document channels of influence and government roles with regard to drug safety; describe institutional contexts in which anti-malarial drug suppliers to the poor operate; review evidence of intervention effectiveness in improving malaria knowledge and in exercising entitlements or rights in health and other sectors, particularly for the poor; and document attitudes and opinions of relevant stakeholders on regulatory processes. Four key issues will be explored in Nigeria under FHS: poverty and vulnerability patterns in selected local areas; citizen empowerment, surveillance, and demand; protecting the poor from low quality anti-malarial drugs through public and private sector engagement; and malaria and drug regulation policies. A cross-sectional study will be conducted in four randomly selected Local Government Areas (LGAs) in Oyo State, while national policy issues will be addressed at the federal level. Relevant literature review will be conducted followed by small scale studies using focus group discussions and key informant interviews. Findings from these studies will be used to develop an intervention, which will test or guide health system changes.

## Consortium activities: a description of the process

This paper reports on activities during the initial phase of the five-year project that aims to inform the global knowledge pool on the research-policy interface in low and middle income countries. First, four key considerations in strengthening evidence-policy linkages in health research were developed by FHS researchers. FHS workshops, held during the inception phase of the research consortium, provided a forum for initial discussions. Key literature was examined at these workshops as well as through electronic communications over a period of six months. The product of this multi-country iterative exploration was an FHS working paper on the evidence-policy interface, which has subsequently been published in peer reviewed literature [16].

Second, the four considerations were applied to each of the six country health system research plans. The focus was identification of features within the research plans that potentially strengthened the research-policy interface and opportunities for improvement based on the four key considerations. Finally, the utility of such an approach for setting research priorities in health policy and systems research was reflected upon and an agenda for empirical work in this field was defined.

## Key considerations for evidence-policy strengthening

The FHS evidence-policy interface conceptual framework highlights three key entry points to the interface; the recognition of the complexity of policy processes; the importance of engaging key stakeholders; and enhancing accountability [16]. These entry points are placed within a developmental context – the importance of this developmental context has been highlighted by FHS [16]. Four key considerations, derived from further work based on this conceptual framework, can provide a lens with which each county concept paper is examined (Table 1). The four key research-policy considerations postulated by FHS are: developmental context; research characteristics; decision making processes; and stakeholder engagement. This section briefly reviews these key concepts while at the same time acknowledging that a detailed discussion and review of each is outside the scope of this paper.

**Table 1:** Key considerations for evidence-policy linkages

• Developmental Context
• Research Characteristics
• Decision Making Processes
• Stakeholder Engagement

### Developmental context

Analysis of the evidence-policy interface requires cognizance of the complex development context within which policy making occurs. Understanding these wider socio-economic dynamics is necessary if health policies are to respond to the needs of the poor by incorporating pro-poor evidence into the policy making process. Four inter-related dimensions to these broader dynamics can be postulated: poverty; vulnerabilities; capabilities; and health shocks. The key points from this complex first key consideration are presented in Table 2.

**Table 2:** Developmental context – a key consideration for evidence-policy.

Cognizance of the complex developmental context within which policy making occurs is crucial to strengthening the evidence-policy interface. Four inter-related dimensions to these broader dynamics can be postulated: poverty; vulnerabilities; capabilities; and health shocks.
• *Poverty *has many manifestations and is not just financially focused – each of these manifestations has a specific relevance to the evidence-policy interface.
• *Vulnerability*, a result of both intrinsic and extrinsic factors, requires detailed analysis in order to understand and adapt pathways from evidence to policy.
• *Capabilities*, both at individual and community level, influence the likelihood of pro-poor health systems based on the evidence base.
• Understanding the devastating effects of *health shocks *on poor individuals and communities is essential if evidence based interventions are to reach the very poor.
Research to elucidate the inter-connections between health shocks and poverty, vulnerability, and capabilities (both at the individual and community levels) may prove critical to strengthening evidence-policy linkages.

*Poverty *can be thought of in absolute or relative terms. Increasingly, the concept is not focused solely on finances (income or resources) or wealth, but includes multiple dimensions, including education, nutrition, health, and other social factors [17]. Each of these aspects of poverty has a particular bearing on the evidence-policy interface. For example, if financial considerations are at the forefront, research examining financial mechanisms to secure access to health services for the poor becomes crucial. However, if the multi-dimensional nature of poverty is appreciated then multiple potential entry points to researching pro-poor health systems emerge [18].

*Vulnerability*, on the other hand, refers to "a dynamic process of negative adaptation in the face of adversity," and is "shaped by prior embodiment of extrinsic factors as well as intrinsic characteristics [19]." Elucidation of these extrinsic factors for particular settings and practical steps to modify them warrant examination in research focused on pro-poor health systems. Gaining an understanding of intrinsic vulnerability characteristics can also allow the refinement of how the evidence base is packaged into action. Understanding either the intrinsic or extrinsic vulnerability factors is no easy task in low-income countries experiencing rapid changes at multiple levels; however such a focus is likely to strengthen evidence-policy linkages.

*Capabilities*, both at individual and community level, influence likelihood of pro-poor health systems. Nussbaum lists these central human capabilities as "life, bodily health, bodily integrity, senses, imagination and thought, emotions, practical reason, affiliation, other species, play, and control over one's environment [20]." Community capabilities, based on these individual capabilities have also been suggested focusing on: preservation of community life within a healthy community; reciprocity within and between communities; emotional inter-dependence; balancing rights and responsibilities; fair distribution based on community values; and institutions that reflect community preferences [20]. Others have emphasized the importance of capabilities related to integration into: economic networks; social networks; and political systems [21,22]. These complex interdependent capabilities are perhaps central to understanding the developmental environment within which evidence is attempting to influence policy making and ultimately policy implementation.

Reports from various low-income settings explicate the negative affect of *health shocks *on poor individuals and communities [23]. The inter-relatedness of poverty, vulnerability, and health shocks are recognized by seeing poverty as "the probability (actual or perceived) that a household will suddenly (but perhaps also gradually) reach a position with which it is unable to cope, leading to catastrophe (hunger, starvation, family breakdown, destitution or death [18]." There is however a relative paucity of empirical evidence on the precise affects of health shocks in these settings [24]. Research that can elucidate the inter-connections between health shocks and poverty, vulnerability, and capabilities (both at the individual and community levels) may prove critical to strengthening evidence-policy linkages. The use of frameworks that allow consideration of multiple developmental factors in health system development may be particularly useful in such endeavors; FHS: Innovations for Equity has developed one such framework [16].

### Research characteristics

Empirical studies on characteristics facilitating incorporation of research into policy are limited in low-income countries. A number of such characteristics have been suggested based on findings mainly from high income countries [25,26]. These include personal contact between researchers and policy makers during the research; timeliness of the research; relevance of the research; research summarization focused on decision maker needs; and inclusion of budgetary considerations within the research [25,26]. Although, the relative importance of these characteristics in low-income countries warrants further testing, it can be postulated that a reality-based world focus in research can increase the chances of influencing policy in low income settings.

Concentrating on operational research – defined as the systematic study, "by observation and experiment, of the working of a system with a view to improvement," is one particular dimension to such a real world focus [27]. Particular areas of exploration that potentially contribute towards such an operational focus include incorporation of a financial dimension and service quality issues within research. These areas of exploration are intricately linked with enhancing accountability within health systems and this has been highlighted as a key entry point to strengthening the evidence-policy interface [16]. Selection of innovative health systems solutions for future oriented research may also capture the imagination of policy makers, thus enhancing the chances of influencing policy making [28,29]. Challenges faced by health systems in low income countries demand innovative solutions; strategic approaches to facilitate diffusion of innovative findings have been developed and tested [30]. These potential solutions may involve multiple sectors outside the traditional health system, thus recognizing the multiple determinants that influence population health status.

### Decision making processes

Understanding the process of decision making in health and where the power to make decisions resides is important. Recent attempts at articulating a framework to assess country-level efforts to link research to action integrate a number of key concepts focusing on integrating decision maker and evidence producer activities [5]. The proposed framework highlights the importance of systemized research prioritization, responding to policy maker priorities, and maintaining close and continuous exchanges between the users and producers of evidence. These interactions reside within a political context of public health policy making that cannot be ignored [31]. Any attempts to either conduct research on particularly favourable or unfavourable subject areas, or the translation of such findings into action need to be cognizant of such a political context.

Key networks and institutions may be central to determining research agendas as well as decision making [32]. Sources for this power may be multiple – knowledge itself, concentrated within such networks and institutions, may be one such source of power. A careful determination of where knowledge resides may thus be a useful undertaking using techniques such as knowledge mapping, which may support knowledge translation [33]. Pathways of influencing such power can then be tested to influence policy outcomes.

### Stakeholder engagement

Stakeholders can be defined as "an individual or group with a substantive interest in an issue, including those with some role in making a decision or its execution [34]." Active participation of a wide range of such stakeholders has been suggested as key to strengthening evidence-policy linkages [16,35]. Potential stakeholders include groups such as: politicians at both the national and local level; resource-allocating authorities; health providers (public and private, formal and informal); beneficiaries (poor and non poor); researchers, international health agencies; civil society; and the general population. Given such a potentially wide array it is essential to consider the whole spectrum of stakeholders specifically in terms of level of interest, power, and support [36].

Conducting such analysis necessitates a systemized approach that allows inclusion of groups that are both intuitive as well as non-intuitive [37]. Such analyses combine multiple methodological approaches and need repeating periodically, as stakeholder perspectives often shift significantly over relatively short periods of time. The ultimate benefit of examining stakeholder positions on health research in a systematic manner is to develop effective systematic strategies to influence these stakeholders to support the transfer of evidence to policy and implementation.

## Findings: six country research agenda analyses

Findings from the analysis of each FHS country research plan with the lens provided by the four considerations in exploring the research-policy interface described above (Table 1) is presented below (summary in Table 3).

**Table 3:** Six country research projects – the four evidence-policy considerations

**Country**	**Health System Research Project**	**C1:Development Context**	**C2:Research Characteristics**	**C3:Decision Making Processes**	**C4:Stakeholder Engagement**
**Bangladesh**	Informal Health Care Systems in Rural Areas	Rural poor depend on informal health sector	Research action focused	Local decision maker perspectives included	Local stakeholders analyzed
**India**	District Level Health Systems Development	Explores health shocks and thus poor focused	Research on practical aspects of decentralization	Decision maker driven	Powerful stakeholders engaged
**China**	Rural New Cooperative Medical Scheme	Focus on health of the rural poor	Research on finance & quality of care	Involves national & local decision makers	Stakeholders engaged throughout research
**Afghanistan**	Influences in Maternal Health Care Seeking	Use of poverty frameworks within research	Research on health seeking inhibitors and enablers	National & international priority	Beneficiary focused research
**Uganda**	Access, Cost, Volume, & Quality of Facilities	Poverty-health connections explored	Operational research focus	Private sector is decision maker priority	Stakeholder workshops planned
**Nigeria**	Effective Malaria Treatment for the Poor	Poverty-malaria linkages explored	Current drug suppliers considered	Malaria is decision maker priority	Non-intuitive stakeholders included

C = Consideration

### Bangladesh: informal health care systems in rural areas

The proposed work incorporates evidence-policy interface considerations in a number of ways. The poor in Bangladesh depend on the informal sector; the chosen subject area as well as the research approach is firmly embedded within a developmental context. The inter-relationship of the informal health sector with individual and community vulnerabilities and capabilities can be elucidated from the proposed research. The effects of health shocks on care-seeking from either the formal or informal health sector can also be explicated by the proposed work. The proposed research is operational in nature and is action focused. Findings will help design future interventions for working with the informal health sector in Bangladesh – thus the process of influencing policy making with research findings can be explored prospectively. Costs and quality of care are integral to the research proposal, which creates a further 'real world' focus of the research. Consideration of how the informal health sector can be incorporated into the health system represents an innovative approach to future health system development.

Project findings on key informal health providers may significantly affect policy making. This decision making process, embedded in a political context, can be examined. For example, a cohort of village "doctors" (non-physicians) was a result of government sponsored training schemes in the past. Study findings on their current role may influence decision-making in relation to these informal health providers. Findings from all local elected representatives (162 elected members of 18 union councils) will provide valuable information on local decision maker perspectives on the health sector. While multiple levels of policy making are recognized in the literature, the more local levels are often ignored – this work attempts to fill this key knowledge gap. In addition, a wide array of local stakeholders is included within the research proposal; many of these stakeholders, for example traditional healers, are non-intuitive stakeholders in formal health system development.

### India: district level health systems development

The proposed work incorporates evidence-policy interface considerations in a number of ways. Key questions surrounding poverty, vulnerability, and capabilities are addressed that attempt to provide answers for decision makers on these issues. The issue of health shocks forms a central focus of enquiry; gaining an understanding of how poverty, vulnerability, and capabilities are related to health shocks may prove particularly valuable. Key aspects of the proposed work focus on practical health system considerations that will inform policy makers. These include: defining who the poor and vulnerable are within a community; establishing health care utilization patterns and financial impacts, especially of the poor; operationalizing possible health insurance schemes; documenting the current supply side environment of health care delivery; and assessment of institutional structures of various possible decentralization mechanisms. This operational focus includes financial consideration, quality issues, innovative approaches, and multiple sector involvement.

The proposed work responds to decision maker priorities – Indian policy makers, specifically in West Bengal are actively exploring decentralization of service delivery to increase responsiveness and accountability to poor and vulnerable groups. The political context of both national and state levels in India is thus appreciated. Institutional capacity of district level management is considered, enriching analysis of the decision making process. An analysis of powerful stakeholders with decision making capacity is a particular focus in the project.

### China: rural new cooperative medical scheme

The proposed work will incorporate evidence-policy interface considerations in a number of ways. The developmental context is central to the work as it is focused on the health of the rural poor. In particular, financing (investigating health shocks) and quality are two central themes addressed, thus highlighting the operational nature of the research.

NCMS is a national priority from a decision maker perspective – operations research on this scheme responds to decision maker needs and the political dimension. Two of the four specific objectives of the research are: to strengthen communication with relevant ministries and involve governmental officials in research; and to disseminate research results to make an equal and pro-poor NCMS. Decision makers (national and local), specific subject experts, as well as researchers will be represented in a National Advisory Panel, thus facilitating continuous exchange of ideas between decision makers and researchers – this can potentially explore the influence of key networks in decision making. The stated goal includes effective communication among actors, and between researchers and stakeholders to ensure the smooth implementation of the project and obtain stakeholders' attention and approval of results. Thus plans are in place to engage key stakeholders throughout the project.

### Afghanistan: influences in maternal health care seeking

Evidence-policy interface considerations are incorporated in the proposed work in a number of ways. Multi-dimensional aspects of poverty and under-development are taken into consideration in the project design. Innovative use of poverty frameworks within the research proposal underlines the importance given to the developmental context. The key research questions focus on individual, household, and community vulnerabilities and capabilities with respect to maternal health, and consider the influence of institutional relationships and actors in a rapidly changing post-conflict context. Research on health seeking inhibitors and enablers provides a 'real world' focus to the work. Key considerations surrounding the effects of finances and quality of care on health seeking behavior are also explored.

Strong political will at multiple levels, both nationally and internationally, exists to address maternal health – thus the proposed work is responding to decision maker priorities. The intervention study incorporates plans for rigorous monitoring and evaluation, and information sharing at all levels, including at the policy development level which will be assessed throughout the life of the project. This places a clear project focus on decision making processes. Attempts are made to engage a wide range of stakeholders (directly and through workshops) within the research itself, focusing on beneficiaries, and such stakeholder engagement is likely to strengthen evidence-policy linkages.

### Uganda: access, cost, volume, & quality of facilities (private/public)

Poverty-health connections are explored in the proposed work, thus incorporating the developmental evidence-policy interface consideration. Qualitative approaches tease out community vulnerabilities, capabilities, and the effects of health shocks. Exploration and testing of empowerment mechanisms and the socio-economic profiling of health service users will provide a particularly strong emphasis on developmental input to the evidence-policy interface. The focus of the research is operational, with a clear mandate to explore costs, quality, and innovations within health system development. In particular, the piloting of innovative approaches in future years has a practical focus – public policy decision makers will be able to utilize such information in deciding on the mix of purchasing options.

National policy in Uganda focuses on private sector involvement as a partner in health service delivery – the proposed work is thus responding to the needs of decision makers. Key informant interviews will include a spectrum of decision makers, providing an understanding of their perspectives. The dissemination plan, including stakeholder workshops, visits to respective ministries, wide ranging publications, and use of the Internet, will also enhance the ability to influence policy.

### Nigeria: effective malaria treatment for the poor

The developmental context is embedded in the work, which focuses on understanding poverty and vulnerability; in particular, poverty-malaria linkages are explored. Capabilities are explored by utilizing the entry point of empowerment for people. An attempt is made to identify major suppliers of anti-malaria drugs, articulate institutional contexts of suppliers, and engage with suppliers from both public and private sectors. This demonstrates the realism-focused nature of the research. A clear focus on quality of care is central to the work, as is the effects of anti-malarial costs on consumption.

Government policies in Nigeria consider poverty eradication and malaria control as high priorities for concerted action – the FHS work thus responds to decision-maker priorities. One of the four key issues explored within the project is malaria control and drug regulation policies (undertaken at the federal, state, and local level) – this places an understanding of policy making processes at the center of the research proposal. A wide array of stakeholders perspectives are considered within the research design with a particular focus on the vulnerable poor at the community level, decision makers at multiple levels, and private providers such as patent medicine vendors. Such wide consideration of stakeholders allows strategies to be formulated for research-policy strengthening.

## Strengthening the interface: cross-country opportunities for improvement

The research proposals from the six countries address many of the suggested considerations for strengthening evidence-policy linkages (Table 3). However, five key areas that may warrant further exploration emerge from a cross country review of current proposals. First, some country proposals may enrich their exploration of the development context with a greater focus on individual and community capabilities [20]. This '*capability*' as opposed to 'vulnerability' focused paradigm may produce findings that can contribute to innovative health systems and is likely to capture decision maker attention. Second, careful attention should be given to the facilitation of *innovations *into health systems either from key informants or from the global knowledge pool. An analysis of common current pathways of innovation diffusion in low and middle income settings may be particularly useful in informing future strategies for effective health system innovation diffusion.

Third, key *networks *which influence decision making need identification and further analyses in many of the countries. Exploration of these key networks involved at the national, district, and community levels, may provide useful information on decision making processes. Informal networks, such as national policy-maker cliques, district level interactions between individuals from different sectors, and influential community-level groups of opinion leader, may especially be a useful avenue of exploration. Fourth, many research proposals have limited focus on institutions involved in the decision making process. These need to be included as power and knowledge are often concentrated within such institutions. Lastly, some research proposals require further clarity on the methodology for engaging key stakeholders. In particular, systematic consideration of the entire range of possible stakeholders is desirable while the iterative nature of stakeholder analysis also needs highlighting.

## Exploring the utility of the approach

Future Health Systems: Innovations for Equity has an opportunity to plan and conduct focused health systems and policy research in some of the largest low-income countries in the world. As a result, careful planning for research, setting priorities, and analyzing the potential for success in both the conduct of research and its utilization for policy change are critical. A review of the planned research is thus instrumental for analyzing the specific potential for innovation within each country and also for contributing to the global pool of knowledge. This paper evaluates these plans for determining how they will contribute to a better understanding of how research evidence influences policy in the health sector – and the value of empirical work in this area – defined as a gap in health policy and systems research [38].

This paper utilizes four key considerations to identify potential leverage points at the evidence-policy interface for future health systems in low-income countries. Such an approach has four main advantages. First, a systematic analysis of research proposals using these key considerations can ensure that all these considerations are addressed or incorporated into the research proposal. Second, the exact meaning, significance, and inter-relatedness of these considerations can be explored within the research itself thus informing the global knowledge pool on the evidence-policy interface. Third, consistent use of the approach by multiple low-income countries for different types of health research can assist cross-country learning. Finally, the ultimate goal of translating evidence into action may be facilitated by such an approach thus enhancing the effectiveness of research endeavors. Resource constraints and low prioritization of health service research in large and small low-income countries make the clarity of this 'research compass' critical.

## Conclusion

Health system research findings in the developing world remain impotent unless translated into public health action through effective public policy-making. Early evaluation of research proposals utilizing the four considerations outlined above provides a potential entry point into analyzing the strength of the research -policy interface. Each research proposal that is analyzed using such an approach should be reported in the public health literature to enhance the global knowledge pool on this critical aspect of health systems research. This will allow the development of a compendium of experiences related to the interface in low and middle income settings for researchers and policy makers to draw upon. The analyses of the six research plans provided in this paper provide a starting point for such explorations.

## Competing interests

The author(s) declare that they have no competing interests. All authors belong to the FHS consortium.

## Authors' contributions

SBS and AAH conceived the work, directed its progression, completed the analyses, and led the writing. GB assisted with the study. All the country team authors (SS; AB; ZZ; BK; OO; and GP) led country level work upon which the paper is based. DP is the primary investigator of the entire Consortium; the work is based upon the research framework he constructed. All authors helped to conceptualize ideas and review drafts of the manuscript. The last author, FHS: Innovations for Equity, recognizes the contribution of all the professionals within the Consortium.
